# Cumulative Impact of Morphometric Features in Schizophrenia in Two Independent Samples

**DOI:** 10.1093/schizbullopen/sgad031

**Published:** 2023-11-04

**Authors:** Rosa Lee-Hughes, Thomas M Lancaster

**Affiliations:** Department of Psychology, University of Bath, Bath, UK; Department of Psychology, University of Bath, Bath, UK

**Keywords:** schizophrenia, psychosis, neuroimaging, meta-analysis, heterogeneity

## Abstract

Schizophrenia and bipolar disorder share a common structural brain alteration profile. However, there is considerable between- and within-diagnosis variability in these features, which may underestimate informative individual differences. Using a recently established morphometric risk score (MRS) approach, we aim to provide confirmation that individual MRS scores are higher in individuals with a psychosis diagnosis, helping to parse individual heterogeneity. Using the Human Connectome Project Early Psychosis (*N* = 124), we estimate MRS for psychosis and specifically for bipolar/schizophrenia using T1-weighted MRI data and prior meta-analysis effect sizes. We confirm associations in an independent replication sample (*N* = 69). We assess (1) the impact of diagnosis on these MRS, (2) compare effect sizes of MRS to all individual, cytoarchitecturally defined brain regions, and (3) perform negative control analyses to assess MRS specificity. The MRS specifically for SCZ was higher in the whole psychosis group (Cohen’s *d *= 0.56; *P* = 0.003) and outperformed any single region of interest in standardized mean difference (Z_MRS>75 ROIS_ = 2.597; *P* = 0.009) and correlated with previously reported effect sizes (P_SPIN/SHUFFLE_ < 0.005). MRS without Enhancing Neuroimaging Genomics through Meta-Analysis weights did not delineate groups with empirically null associations (*t* = 2.29; *P* = 0.02). We replicate MRS specifically for SCZ associations in the independent sample. Akin to polygenic risk scoring and individual allele effect sizes, these observations suggest that assessing the combined impact of regional structural alterations may be more informative than any single cytoarchitecturally constrained cortical region, where well-powered, meta-analytical samples are informative in the delineation of diagnosis and within psychosis case differences, in smaller independent samples.

## Introduction

Global neuroimaging efforts such as the Enhancing Neuroimaging Genomics through Meta-Analysis (ENIGMA) working groups now demonstrate that severe mental illnesses such as schizophrenia and bipolar disorder are associated with alterations in brain structure.^1–4^ These studies demonstrate on average, patients with a psychotic disorder have small-moderate reductions in cortical thickness (Cohen’s *d* up to ~0.5, in the fusiform gyrus) and subcortical volumes (Cohen’s *d* up to ~0.4, in the hippocampus). Moreover, there is a shared structural difference profile between schizophrenia and bipolar.^5^ However, and in most cases, there is considerable overlap between structural metrics for patient groups and control samples, meaning that prospective independent studies require thousands of individuals to reliably detect these effects.^6^ These observations limit ongoing research studies aiming to develop MRI biomarkers and limit utility in potential preclinical, diagnostic, and treatment recovery monitoring applications. The use of any single cytoarchitecturally defined brain region of interest may underestimate patient brain alterations, due to systemic heterogeneity between patients, even within the same diagnostic category and where average differences do not optimally represent any single patient.^7–9^ As brain-wide heterogeneity in patient groups becomes more apparent, multivariate solutions are now aiming to account for inter/intraindividual variability across and between diagnostic categories. For example, the regional vulnerability index,^10–12^ person-based similarity index,^13–15^ and various normative modeling approaches^16–18^ assess a brain-wide profile, based on within-sample deviation and compare to known prior brain alterations. These techniques consider a range of alterations simultaneously and have been successful in delineating case–­control status in severe mental illness.

In an approach inspired by polygenic risk scoring, employed in psychiatric genomics,^19^ we recently created a “morphometric risk score” (MRS) approach, a score reflecting the combined influence of weighted deviations from a sample norm, per individual in the discovery sample.^20^ In this study, we demonstrated that both individuals with a schizophrenia diagnosis and asymptomatic individuals with higher genetic risk for schizophrenia have a higher schizophrenia MRS, compared to controls. By using meta-analysis priors/effect sizes we were able to demonstrate that schizophrenia-associated brain changes were present across a wider cortical landscape, in the absence of significant regional differences. This suggested that schizophrenia case–control differences could be identified in smaller samples than the thousands needed in the ENIGMA discovery samples. This was consistent with prior observations concluding that sample sizes for detecting effects of single nucleotide variants within genome-wide association studies were of the magnitude larger than the sample sizes required to delineate case–control differences using polygenic scoring.^21^ We further demonstrated that ENIGMA schizophrenia effect sizes were essential for successfully delineating case–control differences as MRS generated using no effect sizes/weights did not estimate a comparable group difference.^20^

In the current study, we employ (1) the human connectome project early psychosis (HCP-EP) and (2) an open-access (ds004302) dataset to (a) confirm the utility of the MRS approach in further independent samples; (b) test MRS weight/disorder specificity, and (c) provide confirmatory evidence for the utility of the approach in moderately sized samples. Akin to the polygenic score approach, we further hypothesize that the cumulative impact of all disorder-associated brain alterations would capture a substantial proportion of variance, compared to any single brain region (analogs to any single disorder-associated nucleotide polymorphic variant). We anticipate that robust effect size priors (provided by ENIGMA meta-analysis working groups) will help to parse patient heterogeneity and build individualized models of MRI-based risk on an individual participant basis, in novel datasets. Given the first sample included individuals with diagnoses across the psychosis spectrum (for both nonaffective and affective psychosis), we further aim to explore the shared and unique variance in brain alterations across schizophrenia and bipolar disorder. Here, we aim to further explore the role of (1) psychosis and (2) schizophrenia-specific priors in our MRS estimation, and assess their capability in specifically delineating patients with schizophrenia, considering the shared cortical profile with other affective psychosis.^5,22^ We anticipate that a diagnosis-specific MRS approach may help capture specific clinical features and help to further inform diagnosis. Furthermore, and beyond our prior observations using MRS, we anticipate that the MRS will delineate patients with schizophrenia from healthy controls better than any single region of interest. This approach builds toward a broader initiative in using large, existing MRI data to inform classification in smaller neuroimaging datasets.^23,24^

## Methods and Materials

### Participants (HCP-EP Sample)

Data were collected as part of the HCP-EP Release 1.1, a cross-sectional study of participants between 16 and 35 years of age, focusing on both early nonaffective (including a diagnosis of schizophrenia, schizophreniform, psychosis NOS, delusional disorder, and brief psychotic disorder) and affective psychosis (including a diagnosis of major depression with psychosis or bipolar disorder with psychosis),^25^ assessed within the first 5-year window of the initial onset of psychotic symptoms. This approach enables research to assess a sample with fewer confounds on brain structure such as prolonged medication exposure and chronicity. It is also likely that this time window is where early intervention strategies may be most effective, before the most severe brain alterations occur. The HCP-EP study protocol and consent process were both reviewed/approved by local ethics committees. Written informed consent was obtained from all participants (see study overview https://www.humanconnectome.org/study/human-connectome-project-for-early-psychosis for more details). Intelligence quotient (IQ) was measured with the Wechsler Abbreviated Scale of Intelligence II.^26^ We also implement a series of additional inclusion criteria to closely match the ENIGMA training data, including >18 years old and no mental health diagnosis (DSM-IV: 71.09) or diagnosis of schizophrenia (DSM-IV 295.7) or bipolar disorder (DSM-IV 296.4, 296.44, 296.53, 296.54, 296.89). See table 1 for sample characteristics.

**Table 1.** Descriptive Statistics for the Final Samples Used in the MRS analysis

	Discovery—HCP-EP			Replication—ds004302	
	Bipolar (N = 23)	Healthy control (N = 47)	Schizophrenia (N = 54)	Healthy control (N = 24)	Schizophrenia (N = 45)
Age (years)					
Mean (SD)	24.4 (3.78)	25.2 (4.14)	22.1 (2.74)	40.2 (14.3)	42.5 (10.8)
Median [Min, Max]	24.2 [18.8, 33.3]	24.6 [19.7, 35.7]	21.5 [18.2, 30.5]	40.0 [20.0, 64.0]	42.0 [19.0, 66.0]
Sex					
F	15 (65.2%)	16 (34.0%)	11 (20.4%)	7 (29.2%)	10 (22.2%)
M	8 (34.8%)	31 (66.0%)	43 (79.6%)	17 (70.8%)	35 (77.8%)
Intelligence quotient					
Mean (SD)	108 (16.6)	116 (10.8)	96.4 (16.2)	101 (9.91)	99.8 (9.27)
Median [Min, Max]	113 [53.0, 129]	115 [93.0, 142]	95.0 [59.0, 134]	103 [71.0, 114]	100 [73.0, 116]
Mean surface area (mm^2^)					
Mean (SD)	2510 (265)	2580 (188)	2540 (253)	2540 (244)	2400 (221)
Median [Min, Max]	2440 [2190, 3210]	2580 [2200, 3010]	2580 [1790, 3080]	2550 [2090, 3110]	2410 [1920, 2940]
Mean cortical thickness (mm)					
Mean (SD)	2.86 (0.106)	2.89 (0.0918)	2.79 (0.117)	2.52 (0.105)	2.44 (0.0897)
Median [Min, Max]	2.86 [2.68, 3.06]	2.88 [2.71, 3.08]	2.80 [2.48, 3.05]	2.51 [2.34, 2.80]	2.44 [2.29, 2.62]
Intracranial volume (mm^3^)					
Mean (SD)	1580000 (187000)	1640000 (148000)	1620000 (163000)	1130000 (102000)	1100000 (99200)
Median [Min, Max]	1580000 [1190000, 1950000]	1640000 [1360000, 1910000]	1620000 [1220000, 2030000]	1140000 [922000, 1370000]	1100000 [876000, 1330000]

SD, standard deviation; [Min–Max] represents sample range.

### Participants (ds004302 Replication Sample)

A replication sample was used to compare structural MRI data from 45 patients with schizophrenia (age 42.2 ± 10.80 years, 10 female/35 male) and 24 healthy controls (HCs; age 40.20 ± 14.30 years, 7 female/17 male). Intelligence quotient (IQ) was measured using four subtests of the WAIS III (Vocabulary, Similarities, Matrix reasoning, and Block design).^26^ All participants provided written informed consent for participation, reported in prior publications.^27^ Briefly, all participants were over the age of 18 and younger than 65, with no history of neurological disease/brain trauma, and had no substance/alcohol abuse issues in the prior 12 months. Furthermore, healthy controls were excluded if they had a prior or current psychiatric disorder (or of first-degree relatives) and recent use of psychotropic medication. Detailed sample and preprocessing descriptions are available for this public dataset^27^ available to download at: https://openneuro.org/datasets/ds004302/versions/1.0.1 All data collection study/procedures were approved by the Research Ethics Committee FIDMAG Sisters Hospitallers (Comité de Ética de la Investigación de FIDMAG Hermanas Hospitalarias) and complied with its ethical standards on human experimentation, where all participants provided informed consent.

### T1w Acquisition and Preprocessing

For study 1, the structural MRI data were collected across four sites in the United States: Indiana University (*n* = 60), Brigham and Women’s Hospital (*n *= 17), McLean Hospital (*n *= 33), and Massachusetts General Hospital (*n *= 14). T1 weighted MRI scans were conducted using Siemens MAGNETOM Prisma 3-Tesla (3T) scanners, with T1-weighted MPRAGE with a slice thickness = 0.8 mm, field of view = 256 mm, and voxel volume = 0.8 × 0.8 × 0.8 mm: flip angle = 8°. For the replication study, images were acquired with a 3T Philips Ingenia scanner (Philips Medical Systems, Best, The Netherlands). High-resolution T1-weighted images were acquired with a fast field echo sequence (TR/TE = 9.90/4.60 ms; Flip angle = 8°; 1 mm^3^ isotropic voxel collection of 180 slices and a field of view = 240 mm). All imaging data (Studies 1 and 2) from the MRI scans were preprocessed through FreeSurfer version 7.1.1 (https://surfer.nmr.mgh.harvard.edu/fswiki/ReleaseNotes). Cortical thickness (mm^2^) of 34 cortical regions and the volume (mm^3^) of 7 subcortical regions were estimated using the processing and reconstruction method outlined by Fischl et al^28^ to segment and label cortical and subcortical volumes based on cytoarchitectural boundaries via the Deskian Killiany atlas.^29^

### MRS Calculation

Measurements for 75 bilaterally averaged regions of interest (7 subcortical volume, 34 cortical thickness, 34 cortical surface area) were calculated. Each of the 75 regions of interest was deconfounded for sex, age, and global size (mean thickness for thickness metrics, mean surface area for surface metrics, total ICV for subcortical measurements). These residuals were z-transformed/rescaled to allow equal weighting across all metrics and permit standardized outlier detection. We employed an MRS approach, as previously described. Briefly, for each subject, we estimated the absolute mean product of the ENIGMA weight and the normalized region of interest, when the sign of the two estimates was congruent. All residual region of interest units were adjusted using ComBat^30^ to minimize confounding from site effects on MRI metrics.^31^ As the HCP-EP sample consisted of individuals diagnosed with a range of psychotic disorders, we utilize ENIGMA effect sizes/weights that reflect the transdiagnostic nature of this sample (see Participants). We therefore used the weights estimated from both schizophrenia and bipolar disorders.^1–4^ We created three experimental MRS, the first principal component of shared variance between schizophrenia and bipolar (SCZ + BD) and the unique variance for schizophrenia and bipolar (SCZ-unique, BD-unique). For a fourth MRS, we generated a negative control analysis/null MRS (H0) where no ENIGMA weights were used, aiming to assess the utility of the ENIGMA weights in shaping the MRS specificity.


$$\begin{array}{l} \varphi \;=\;\overset{N}{\underset{i=1}{\sum }}\theta \chi \;\left\{if\;\theta \chi >0\right\} \end{array}$$

Equation 1. The MRS formula; ***φ*** is an individual’s MRS,  χ is a brain region of interest; θ is the corresponding, established brain region effect size (standardized mean difference; SMD) from prior ENIGMA disease working group meta-analysis.

### Power Calculation

We estimated power using the “pwr” library in the R environment.^32^ In our prior study, we observed a minimum standardized mean difference (SMD) = 0.61 schizophrenia MRS between patients and healthy controls.^20^ Based on this effect size, we had >90% power (alpha = 0.05) to detect a comparable effect in a sample of 47 healthy controls and 77 psychosis patients.

### Statistical Analysis

Each dependent variable (individual region of interest, MRS metric) was regressed against diagnostic status, in a linear mixed model regression analysis, where age, sex, intelligence quotient, intracranial volume, mean cortical thickness, and surface area as additional fixed effects and site as a random effect (see table 1). For our linear mixed models, we employed outlier labeling and detection,^33^ defining and removing outliers using the interquartile range outlier labeling rule (1.5 × interquartile range [Q3 − Q1]). This approach dynamically removed individual MRS data points to minimize the impact of outlier data points. Case–control difference brain maps from the present study were correlated with prior effect size brain profiles using p-spin testing, where surrogate null models preserve the spatial autocorrelation of parcellated ROI effect sizes.^34,35^ The replication sample was analyzed using the same formula without the random effect, as it was all collected at a single site. Last, we generated a logistic regression model classifier in the “caret” package^36^ to assess the accuracy on the HCP-EP MRS model in our out-of-distribution (ODT) replication dataset.

## Results

### Main MRS Group Effects

**Fig. 1.**
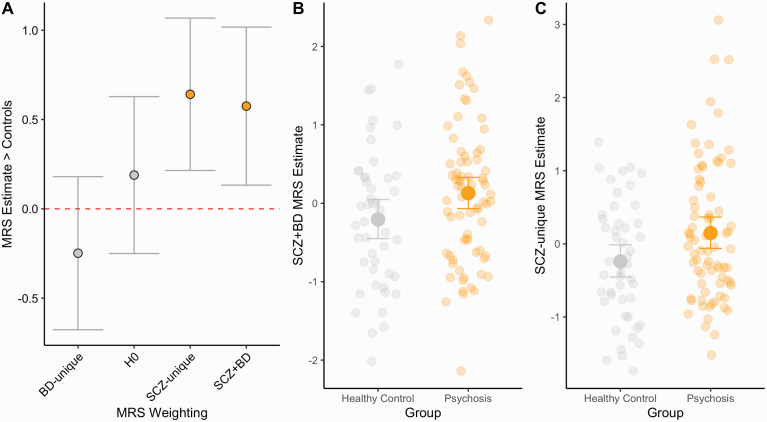
(A) Beta coefficients and 95% confidence intervals for all MRS regressed against diagnosis (psychosis > controls). (B and C) Individual data group differences in SCZ + BD and SCZ-unique MRS, comparing healthy controls to individuals with a psychosis diagnosis. Y-axis reflects adjusted MRS estimate for SCZ + BD. Error bars reflect 95% bootstrapped confidence intervals.

**Fig. 2.**
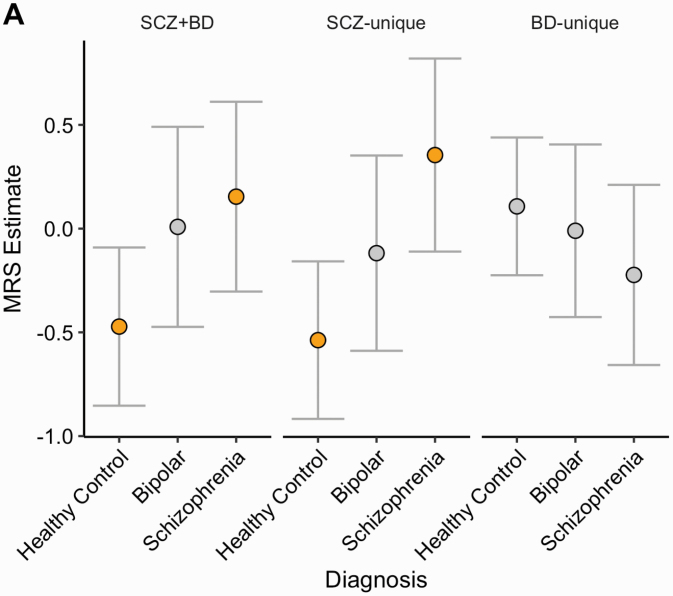
Adjusted SCZ + BD, SCZ-unique, and BD-unique MRS mean estimates and 95% confidence intervals, grouped by diagnosis subtype. Significant (& P-corrected) MRS-wide diagnostic differences between Healthy Control vs. Schizophrenia for SCZ+BD and SCZ-unique.

Individuals with a psychosis diagnosis had a higher SCZ + BD and SCZ-unique MRS than the healthy control sample (Cohen’s *d* = 0.48, 0.56, P_FDR_ < 0.05, see figures 1A–C, table 2), adjusted for confounds. We observed no association between these MRS and global measures, suggesting the association was not explained by global reductions in intracranial volume, mean cortical thickness, or surface area. To ensure that the SCZ + BD/SCZ-unique MRS were specific in shaping the MRS, we further created an MRS which considered no weights (H0-MRS), calculated as the product of the normalized/adjusted region of interest residuals. H0-MRS was also not associated with diagnosis (*P* = 0.401, figure 1A; table 2). Furthermore, a comparison of effects sizes demonstrated that the degree of association between SCZ + BD and SCZ-unique MRS with psychosis diagnosis was higher than H0 (*t*_SCZ+BD_ = 1.75, *P* = 0.04; *t*_SCZ-UNIQUE_ = 2.28, *P* = 0.01, respectively), assessed with a test of differences between two dependent correlations (see figure 1A). We observed no difference BD-unique-MRS between the groups (*P* = 0.345). A post hoc analysis demonstrated that the SCZ + BD and SCZ-unique MRS were higher in the schizophrenia group compared to the healthy controls (*P*_ADJUSTED_ = 0.003, *P*_ADJUSTED_ = 0.050, respectively), but no evidence for difference between healthy controls and bipolar cases (*P*_ADJUSTED_ > 0.2, in all three MRS cases) or between schizophrenia and bipolar (*P*_ADJUSTED_ > 0.3, in MRS both cases), see figure 2; table 3 for adjusted SCZ + BD MRS means by diagnosis sub-group, MRS adjusted group differences highlighted in orange. To assess if MRS group differences could be explained by medication, we included lifetime antipsychotic medication exposure/chlorpromazine equivalent (*M* = 420 ± 207 [100–1000 mg], as previously calculated^37^) as covariates, which did not affect any group differences for either MRS in the wider sample or correlate with MRS in a patient only group (*P*s > 0.1, in all cases).

**Table 2.** Coefficients Summary for Fixed Effects of Each MRS, Controlling for Site Random Effects and Adjusted for Outliers

MRS	BETA	SE	DF	P-CORR
SCZ + BD	0.575	0.226	114.000	0.024
SCZ-unique	0.642	0.218	107.435	0.016
BD-unique	−0.248	0.219	112.000	0.345
H0	0.189	0.224	114.000	0.401

BETA, coefficient of slope between healthy controls and psychosis patients; SE, standard error of beta coefficient; DF, degrees of freedom; P-CORR reflects adjustment via false discovery rate (FDR).

**Table 3.** Post Hoc, Diagnosis-Specific Summary for Fixed Effects of Each MRS, Controlling for Site Random Effects and Adjusted for Outliers

MRS	CONTRAST	BETA	SE	DF	P-CORR
PC1	Bipolar–healthy control	0.481	0.291	48.977	0.234
	Bipolar–schizophrenia	−0.146	0.32	50.655	0.892
	Healthy control–schizophrenia	−0.626	0.264	112.87	0.050
SCZ-unique	Bipolar–healthy control	0.419	0.284	57.52	0.309
	Bipolar–schizophrenia	−0.473	0.318	56.86	0.304
	Healthy control–schizophrenia	**−**0.892	0.266	114.64	**0.003**
BD-unique	Bipolar–healthy control	−0.118	0.249	53.191	0.884
	Bipolar–schizophrenia	0.213	0.286	46.405	0.738
	Healthy control–schizophrenia	0.33	0.238	109.03	0.349

BETA, coefficient of slope between each diagnostic contrast; SE, standard error of beta coefficient; DF, degrees. P-CORR reflects adjustment via false discovery rate (FDR).

### MRS Effect Size Comparisons

**Fig. 3.**
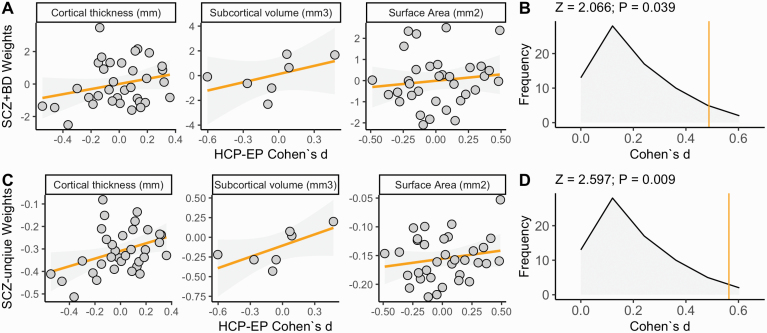
(A and C) Scatter plots reflecting similarity between (A) SCZ + BD and (C) SCZ-unique region of interest loadings and standardized mean differences between psychosis patients and healthy controls in the HCP-EP, corrected for covariates. Individual data points reflect region of interest effect sizes, this correlation was validated with P-spin/shuffle tests. Grey shading indicates 95% confidence interval of regression slope. (B and D) Frequency of individual region of interest effect sizes for 75 regions. Vertical lines intersecting x-axis reflect the MRS standardized mean difference (SMD = 0.48, 0.56, respectively). X-axis reflects the SMDs (reported in absolute units, rather than signed) to scale the comparison with the MRS effect size.

We then compared the main effect size against the between-group effect size for each region of interest individually, using the same linear mixed model formula. No single region of interest was significantly reduced/increased in the psychosis group after correction for multiple comparisons (lowest *P*_UNCORRECTED_ = 0.004; inferior temporal thickness). This was expected as the statistical power to detect individual regional ENIGMA effect sizes in this sample (*N* = 47/77) range from 2% to 51% power for schizophrenia and 0.1%–27% power for bipolar disorder. However, the individual regions of interest for patient/control standard mean differences were correlated with the SCZ + BD and SCZ-unique weights suggesting that the profile of structural alterations was present, just underpowered at the level of individual region of interest (linear mixed models with metric) volume, thickness, surface area as random effect, *r* = 0.45, *P* = 3.3 × 10^−5^, see figures 3A and C. This was supported by metric-specific correlations between the HCP-EP effect sizes and SCZ-ENIGMA effect sizes for cortical thickness (*P*_SPIN_ = 0.003) and subcortical volume (*P*_SHUFFLE_ = 0.006), but not surface area (*P*_SPIN_ = 0.169) or any BD-ENIGMA effect size (*P* > 0.1, in all cases). We then compared all the regions of interest for psychosis versus control SMDs (*N* = 75) to our SCZ + BD and SCZ-unique MRS SMD (*N* = 2, figures 3B and D), demonstrating that SCZ + BD and SCZ-unique MRS psychosis-associated SMD were larger than all individual regions of interest effect sizes differences (Z_SCZ+BD MRS > 75 ROIS_ = 2.066, *P* = 0.039; Z_SCZ-UNIQUE MRS > 75 ROIS_ = 2.597, *P* = 0.009).

### Replication Sample (ds004302)

**Fig. 4.**
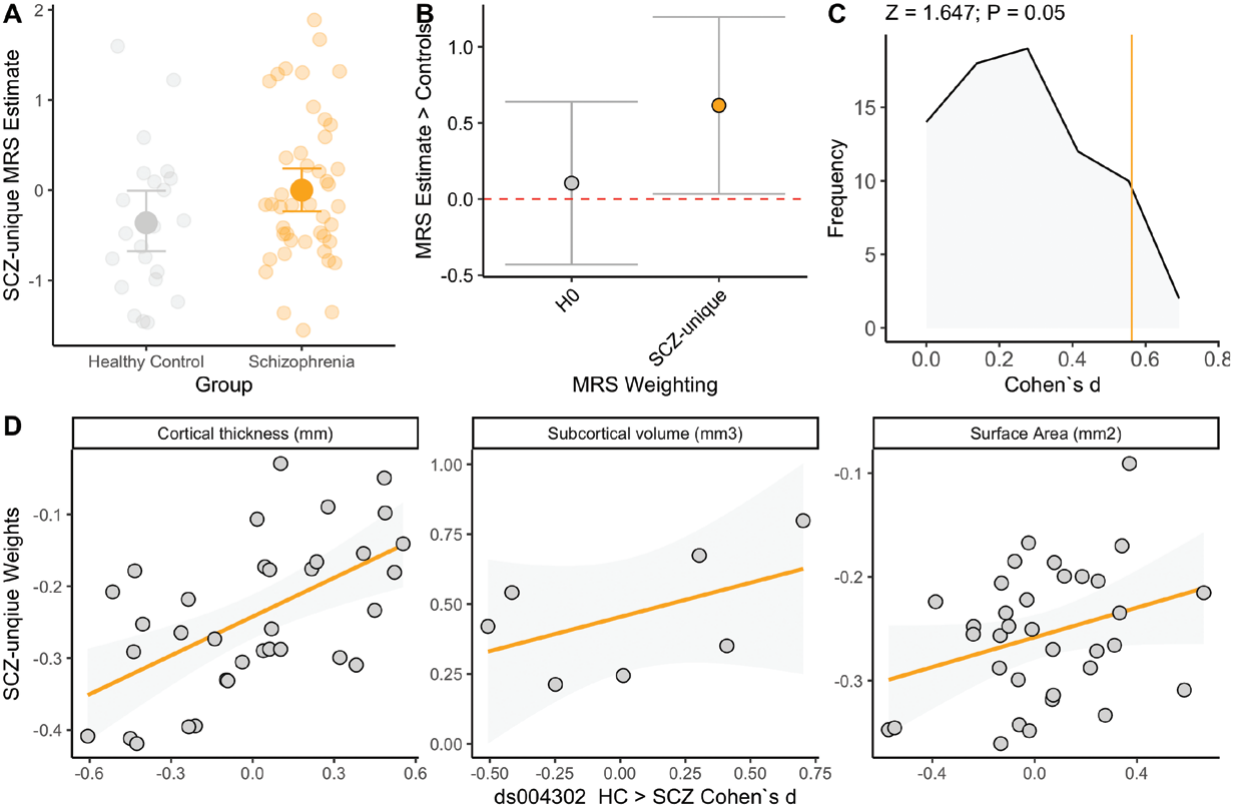
(A) Individual data group differences in SCZ-unique MRS, comparing healthy controls to individuals with a schizophrenia diagnosis. Y-axis reflects adjusted MRS estimate for SCZ + BD. Error bars reflect 95% bootstrapped confidence intervals. (B) Beta coefficients and 95% confidence intervals for SCZ-unique and H0 MRS regressed against diagnosis (schizophrenia > controls). (C) Frequency of individual region of interest effect sizes for 75 regions. Vertical line intersecting x-axis reflects the MRS standardized mean difference (SMD = 0.56). X-axis reflects the SMDs (reported in absolute units, rather than signed) to scale the comparison with the MRS effect size. (D) Scatter plots reflecting similarity between SCZ-unique region of interest loadings and standardized mean differences between schizophrenia patients and healthy controls in ds004302, corrected for covariates. Individual data points reflect region of interest effect sizes. Grey shading indicates 95% confidence interval of regression slope.

Individuals with a schizophrenia diagnosis had a higher SCZ-unique MRS than the healthy control sample (Cohen’s *d* = 0.56, *P* = 0.042, see figure 4A), adjusted for confounds. A comparison of effects sizes demonstrated that the degree of association between SCZ-unique MRS with schizophrenia diagnosis was higher than H0 (*t*_SCZ-UNIQUE_ = 2.3, *P* = 0.02), assessed with a test of differences between two dependent correlations (see figure 4B). The SCZ-unique MRS schizophrenia-associated SMD was similarly larger than all individual regions of interest effect sizes differences (Z_SCZ-UNIQUE MRS > 75 ROIS_ = 1.67, *P* = 0.050; figure 4C). The overall profile of structural alterations was correlated with the SCZ-unique weights, partial *r* = 0.47, *P* = 2.1 × 10^−5^, driven by cortical thickness (*P*_SPIN_ < 0.001), but not volume or surface area (*P*_SPIN_ > 0.1, in both cases), see figure 4D. Last, we assessed the predictive capacity of HCP-EP model on the replication data, demonstrating that the model had a classification accuracy of 69.56% [95% CI: 0.57–0.80].

## Discussion

Disorders across the psychosis spectrum are associated with morphological differences across the brain, with heterogeneity in mean differences within (across the brain) and between diagnoses.^1–9,14,38^ While these effects have been established in large samples, there is a limited, but growing body of work aiming to assess how these morphometric difference features can be integrated and utilized for downstream applications such as prediction, diagnosis, and monitoring recovery. Here, we expand our prior line of research to confirm that an MRS approach is effective in delineating case–control differences in small-moderate ENIGMA-independent samples, in the absence of regional alterations assessed by typical univariate assessment. The standardized mean difference (SMD) for SCZ + BD and SCZ-unique MRS between patients and psychosis group were moderate (SMD = 0.48, 0.56) and higher than any individual effect size estimated at a single region of interest. While this is comparable to the effect sizes we observed in our initial study,^20^ we suggest these effect sizes are treated with caution until observed in larger samples. Furthermore, this association was corrected for global measurements (intracranial volume, mean cortical thickness, and surface area), suggesting that the MRS reflected individualized patterns of risk, rather than systematic and ubiquitous reductions in these global metrics. We therefore suggest that akin to polygenic scoring, a cumulative assessment of individual risk, weighted by prior knowledge of known effects may capture more variance in case–control differences than single region of interest (or loci) individually.^39^ The MRS standardized mean differences were larger than those of any single regions of interest, empirically demonstrating the cumulative impact of MRI features can be observed in the absence of individual cytoarchitectural alterations and is a critical development towards the use of established data to inform, smaller, more clinically orientated studies.^24^ We also further provide evidence that the prior effect sizes, derived from large case–control meta-analysis is a critical aspect of the estimation, as the absence of weights failed to delineate case–control status. This suggests the MRS approach may be a useful approach for monitoring brain health in small-moderate samples, compared to the large samples required to detect robust, regional difference estimates. This provides confirmatory evidence that the specific ENIGMA weight type is critical in delineating case–control status and novel evidence that unique variance assigned to SCZ was instrumental in delineating within the psychosis spectrum, between schizophrenia and controls, but not bipolar disorder. This development indicates that the MRS may be useful for delineating between diagnosis with a shared neurobiological profile.^5,22^ We did not observe any bipolar MRS associations, but this could be explained by the proportionally larger sample of schizophrenia compared to bipolar patients.

We note that other multivariate approaches such as the regional vulnerability index (RVI),^10,12^ person-based similarity index (PBSI)^13–15^ and normative modeling^15,23^ also demonstrate the capacity to delineate case–control differences, however, these require that each participant adheres to an established cortical profile rather than an independent consideration of all regional effect sizes. We further note that the ENIGMA “dot-product” also produces an averaged product^10,12^ but our MRS has an additional statement about effect size congruency. We acknowledge that future studies that compare the validity, reliability, and specificity of these metrics may be useful in optimizing future multivariate solutions. We had no a priori predictions about which self-report/symptom profile our MRS would relate to, as each individual MRS may relate to independent alterations across distinct cortical networks, where two individuals may have higher MRS, representing cumulative alterations across different network topologies, compared to psychotic symptoms which appear to have comparatively specific morphological correlates.^40,41^ Future studies in larger cohorts should take dimension reduction/data-driven approaches to establish the clinical characteristics related to an increased MRS.

Our findings should be considered with several limitations. First, we cannot ascertain whether the MRS reflects a genetic antecedent and/or a product of environmental influence as genetic data was not available for these samples. While our initial report suggested that individuals with higher genetic risk for schizophrenia also had a higher MRS, it remains unknown how genetic risk may shape the MRS across the psychosis spectrum. While patients in the HCP-EP were assessed within the first 5 years of diagnosis and we observed no association with antipsychotic medication, the MRS may be partly explained by confounding from downstream factors such as age of onset/disease duration. Second, we must consider that our prediction is shaped by the characteristics of the discovery data we employ as priors. Therefore, the MRS may underperform in groups of individuals that were disproportionally unrepresented in discovery samples, which may exacerbate inequality in the progress of medical research. Third, we acknowledge that our MRS was estimated using a coarse cytoarchitectural atlas comprised of a small number of brain regions. In the future, more sophisticated discovery priors, such as vertex-wise maps or multimodal MRI data fusion (eg, with diffusion-weighted MRI data^11,42–44^) may be useful in improving risk morphological profiles. It would also be prudent to assess the MRS techniques in multi-site/leave-one-out analyses to estimate generalizable mean differences. Fourth, one difference between our previous study and the current research is that the present work studied a heterogeneous patient sample. As we were assessing patients across the psychosis spectrum, the ENIGMA weights that were captured shared variance performed better than modeling schizophrenia and bipolar MRS separately, however, we acknowledge that a larger nonaffective sample size would have been useful in delineating unique variance. Future studies that are adequately powered to explore MRS-based differences across the psychosis spectrum (eg, schizophrenia vs bipolar disorder) may be useful in exploring neural correlates that are distinct between these disorders.^45^ Last, and at present—we can only estimate MRS for groups of individuals collected via the same MRI protocol, making individual MRS assessment a challenge for personalized therapy/care. We hope that MRS may offer clinical/diagnostic utility beyond the context of the sample from which they are acquired. For example, as recent advances in normative modeling and established brain charts become more available,^46^ we may be able to reliably calculate MRS for specific individuals via comparisons to independently acquired reference data. Our initial ODT classification test demonstrated ~70% accuracy in our replication sample—comparable to ODT predictions of schizophrenia using neuroimaging in combination with sophisticated machine learning tools,^47,48^ suggesting that while it requires further development before clinical use, it offers a parsimonious, scalable and transparent solution to classification using multivariate MRI features, which may benefit from larger/more diverse training datasets and cross-modal integration.

In conclusion, we provide confirmatory evidence that the cumulative impact of sub-threshold alterations in brain structure can be used to delineate case–control differences with moderate effect sizes. We speculate that the MRS approach can be employed in a scenario where well-established alterations have previously been reported in an independent dataset. We anticipate that our MRS approach will help to progress future efforts using neuroimaging to further refine individual solutions for diagnostic prediction, classification, and disease monitoring helping to parse and understand the heterogeneity in morphological alterations reported across the psychosis spectrum.^49^
